# Novel Peptide Analogues of Valorphin-Conjugated 1,8-Naphthalimide as Photodynamic Antimicrobial Agent in Solution and on Cotton Fabric

**DOI:** 10.3390/molecules29225421

**Published:** 2024-11-17

**Authors:** Desislava Staneva, Petar Todorov, Stela Georgieva, Petia Peneva, Ivo Grabchev

**Affiliations:** 1 Department of Textile, Leathers and Fuels, University of Chemical Technology and Metallurgy, 1756 Sofia, Bulgaria; 2 Department of Organic Chemistry, University of Chemical Technology and Metallurgy, 1756 Sofia, Bulgaria; pepi_37@abv.bg (P.T.); petenceto_2@abv.bg (P.P.); 3 Department of Analytical Chemistry, University of Chemical Technology and Metallurgy, 1756 Sofia, Bulgaria; st.georgieva@uctm.edu; 4 Faculty of Medicine, Sofia University “St. Kliment Ohridski”, 1504 Sofia, Bulgaria

**Keywords:** light-activated, peptides, valorphin, cotton fabric, 1,8-naphthalimide, antimicrobial

## Abstract

For the first time, N-modified analogues of VV-hemorphin-5 (Valorphin) were synthesised and conjugated with three different 4-substitured-1,8-naphthalimides (H-NVal without substituent, Cl-NVal with chloro-substituent, and NO_2_-NVal with nitro-substituent). Cotton fabric was modified with these peptides by soaking it in their ethanol solution, and the colourimetric properties of the obtained fabric were measured. The fluorescent analysis shows that peptide immobilisation on a solid matrix as fabric decreases the molecule flexibility and spectrum maxima shift bathocromically with the appearance of a vibrational structure. The peptides’ contact antimicrobial activity, and the resulting fabrics, have been investigated against model Gram-positive *B. cereus* and Gram-negative *P. aeruginos* bacteria. For the first time, the influence of light on bacterial inactivation was investigated by antibacterial photodynamic therapy of similar peptides. Slightly more pronounced activity in liquid media and after deposition on the cotton fabric was obtained for the peptide containing 4-nitro-1,8-naphthalimide compared to the other two peptides. Immobilisation of a peptide on the surface of fibres reduces their antimicrobial activity since their mobility is essential for good contact with bacteria. Cotton fabrics can be used in medical practice to produce antibacterial dressings and materials.

## 1. Introduction

The growing interest in products with antimicrobial and self-cleaning properties, such as sportswear, home textiles, underwear, and protective clothing, is closely tied to improving people’s quality of life and well-being. This interest is particularly pronounced in medical practice, where pathogen-preventing textiles are instrumental in reducing nosocomial infections, a leading cause of post-surgical complications and mortality [1]. Much attention is also paid to the functionality of children’s clothing, which, in addition to aesthetics, must provide comfort and protection. Antimicrobial clothing for children has specific requirements that must be considered in terms of use and safety [2,3].

As natural products, cotton materials have found widespread everyday use. Due to their high hygroscopicity, they retain moisture, which, under certain conditions, makes them a suitable environment for developing pathogenic microorganisms that can cause various diseases. Therefore, to prevent this negative tendency, they have to be treated with substances with antimicrobial activity.

The resistance of pathogenic bacteria and the increase in knowledge of the role of the microbiome inside the human body and outside on the skin determine the need to search for new antimicrobial agents and strategies to prevent infectious diseases. Various substances with antimicrobial properties have been applied to treat textile materials, but those of natural origin are of current interest. These include antimicrobial peptides, which are part of the immune defense of animals, insects, and humans [4,5]. They are much less susceptible to the development of microbial resistance and can be further functionalized. Antimicrobial peptides are positively charged, amphipathic structures of great diversity and short molecular length. They exhibit a broad spectrum of activity against Gram-positive and Gram-negative bacteria, viruses, and parasites over a wide pH and temperature range. Recently, they have been defined as “natural antibiotics” [6,7].

Since the landmark discovery of antimicrobial peptides in the 1970s, many studies have been undertaken to understand their preparation and the intricate mechanisms of their action. Ribosomal synthesis, a crucial part of the innate immune system, is involved in their synthesis. However, it is important to note that the process is not limited to this, as it also occurs through the proteolytic degradation of specific proteins within the organism, highlighting the complexity of the process [8,9]. Each protein molecule encodes enormous information in its primary, secondary, tertiary, and quaternary structures, and the energy required to build up such a complex molecule should be well-spent on more than one task [10]. So, process optimization and recycling are natural clever tricks for using the complex protein structure for many purposes. A typical example of this phenomenon is hemoglobin. Many studies demonstrated that hemoglobin and its derived fragments have various biofunctionalities [11,12]. P. Mak et al. revealed that native hemoglobin and other heme-containing proteins do not exhibit antimicrobial properties. However, the partially unfolded polypeptides, which do not contain heme after hemoglobin hydrolyzation, have antimicrobial activity. The authors proposed naming these proteins hemocedins, in contrast to, e.g., hemorphins displaying opioid-like activity [13].

Our recent research has demonstrated that *N*- and C-modified hemorphin analogues, obtained with solid-phase peptide synthesis, exhibit antiviral and antibacterial activity in solution and on cotton fabric [14,15]. When labelled with a fluorophore, the peptides offer a practical method for regulating their antimicrobial activity under the influence of light [16,17,18]. We have also found that hemorphines conjugated with rhodamine increase their antimicrobial activity against Gram-positive and Gram-negative bacteria under light. Applying these antimicrobial peptides to textile materials is a promising tool for enhancing biocompatibility and providing antibacterial effects against clinical multiresistant bacteria [19]. However, newly synthesized biologically active substances are often water-insoluble because they do not contain ionic substituents. Therefore, alternative methods must be sought for their uniform application and connection with the functional groups of the textile materials [20]. A solution to this problem is treating the fabric in an organic solvent environment rather than water.

This study aimed to synthesize new antimicrobial peptides derived from valorphin modified with 1,8-naphthalimide fluorophore with different substituents in four positions in the molecule, which influenced their interaction with light. We have modified cotton fabric with these peptides, and its colourimetric properties have been measured. The contact and photodynamic antimicrobial activity of the new peptides and the resulting fabrics have been compared.

## 2. Results and Discussion

### 2.1. Design and Synthesis of New Peptides

**Scheme 1:**
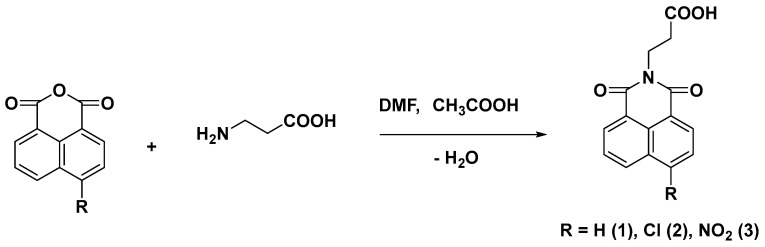
Synthesis of 1,8-naphtalimide-β-alanine (**1**), 4-chloro-1,8-naphtalimide-β-alanine (**2**) and 4-nitro-1,8-naphtalimide-β-alanine (**3**).

For the preparation of new N-modified analogues of VV-hemorphin-5 (Valorphin) with 1,8-naphthalimide (H-NVal, Cl-NVal, NO_2_-NVal), firstly, it needs to be synthesized. 1,8-naphtalimide-β-alanine (**1**), 4-chloro-1,8-naphtalimide-β-alanine (**2**), and 4-nitro-1,8-naphtalimide-β-alanine (**3**) [21] (Scheme 1).

The compounds **1**–**3** are potential fragments for pharmaceutical and many other research and development applications, such as those incorporated in biomolecular systems and are capable of recognizing and responding to external stimuli such as ligand binding, a change in pH or temperature, or absorbance of a photon, etc. [22,23,24,25]. The starting compounds **1**–**3** used to obtain the desired compounds H-NVal, Cl-NVal, and NO_2_-NVal were synthesized from corresponding 1,8-naphthalic anhydride under the influence of β-alanine in the medium of *N*,*N*-dimethylformamide and acetic acid (Scheme 1) to give the corresponding 4-substituted/unsubstituted 1,8-naphtalimide-β-alanine (**1**–**3**).

The N-modified analogues of VV-hemorphin-5 with 1,8-naphthalimide (H-NVal, Cl-NVal, NO_2_-NVal) were prepared by solid-phase peptide synthesis (SPPS)-Fmoc (9-fluorenylmethoxy-carbonyl) strategy using TBTU (2-(1H-benzo-triazole-1-yl)-1,1,3,3-tetramethyluroniumtetrafluoro-borate), an efficient peptide coupling reagent (Figure S1) [26,27]. The SPPS was based on the reaction of 1,8-naphtalimide-β-alanine (**1**), 4-chloro-1,8-naphtalimide-β-alanine (**2**), or 4-nitro-1,8-naphtalimide-β-alanine (**3**) with N-terminal amino group of Val-Val-Tyr-Pro-Trp-Thr-Glu heptapeptide directly on the resin. After cleavage of the product from Rink-Amide MBHA Resin by TFA/TIS/H2O to give the peptide compounds H-NVal, Cl-NVal, and NO_2_-NVal, the 1,8-naphthalimide-peptides were purified from crude product by semi-preparative HPLC with a C18 column. The molecular weights were determined using HRMS (ESI). The structures of the obtained compounds were proven via UV-Vis, FTIR, and NMR (^1^H, ^13^C, NOESY, COSY) spectra (Figures S2 and S3).

We synthesized the new 1,8-naphthalimide-conjugated peptide derivatives (H-NVal, Cl-NVal, NO_2_-NVal) by replacement of the glutamine (Gln) at the C-terminus, contained in natural Valorphin (heptapeptide: Val-Val-Tyr-Pro-Trp-Thr-Gln) with glutamic acid (Glu), on the one hand, to estimate the influence of Glu on the antimicrobial activity of the peptides, and on the other hand, the binding of the peptide molecules to the textile material. Aiming to additionally prove the importance of 4-substituted/unsubstituted 1,8-naphtalimide-β-alanine residues and antimicrobial potency, we introduced the 1,8-naphtalimide-β-alanine (**1**), 4-chloro-1,8-naphtalimide-β-alanine (**2**), and 4-nitro-1,8-naphtalimide-β-alanine (**3**) to the N-side of VV-hemorphin-5 to obtain the peptides H-NVal, Cl-NVal, and NO_2_-NVal. 1,8-Naphtalimide-containing compounds have long been proven to have many therapeutic applications, such as antiviral, antibacterial, antiprotozoal, anticancer, etc. [28,29].

### 2.2. NMR Characterization of the Peptides

The following 2D experiments of NMR analysis were conducted: COSY (Correlated Spectroscopy) and NOESY (Nuclear Overhauser Enhancement Spectroscopy). The COSY displays [1H,1H]-correlations due to scalar (through-bond) couplings and NOESY used for short peptides characterization. The efficiency of the coherence transfer vastly decreases with increasing linewidth, which is related to the molecular weight. By comparing some fingerprint regions about NHi+1-αHi peaks of COSY and NOESY, intraresidual and sequential NH-αH peaks can be distinguished and determine the exact position in the amino acid sequence [30,31,32]. Positions of cross peaks in the COSY are characteristic of the amino acids: αH-βH of Val, Glu, and Pro: 1.7 to 2.6 ppm; all non-labile, non-aromatic sidechain protons except those from βH-γCH_3_ of Thr, δH-δH of Pro: 1.0 to 4.0 ppm; αH-βCH of Phe, Tyr, and Trp: 2.8 to 4.0 ppm; αH-βH of Thr, δH-δH of Pro: 4.0 to 5.0 ppm; aromatic ring protons: 6.2 to 8.0 ppm; backbone NH-αH (fingerprint-region): 6.7 to 9.0 ppm.

### 2.3. Modification of Cotton Fabric with the Peptides

The cotton fabric was modified by soaking with an alcohol solution for each of the newly synthesized peptides. This method is preferred because the peptides are insoluble in water but well soluble in ethanol. The advantage of this solvent is that it evaporates quickly after being applied to the fabric [20]. The required amount of solvent is small and not toxic at these concentrations, ensuring the safety of the process. Since the peptides are insoluble in water, their leaching from the fibre surface in the water bath is not observed when the fabrics are rinsed with water and washed with detergent. The obtained fabrics were named H-NValC, Cl-NValC, and NO_2_-NValC. Irradiation with UV light at 365 nm shows the uniform distribution of peptides on the cotton fabrics.

### 2.4. Colourimetric Analysis of the Studied Fabrics

**Figure 1:**
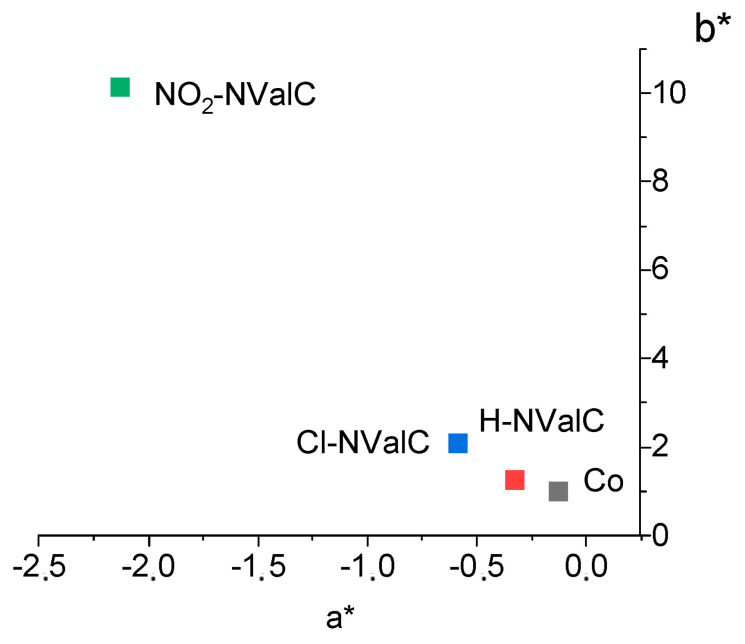
The red/green (a*) and yellow/blue (b*) colour coordinates of the pristine fabric and the fabrics modified with the peptides.

The colour parameters of the fabrics have been characterized by main CIELab coordinates (L*a*b*). Figure 1 shows the two colour coordinates, a* and b*, which correspond to the red/green and yellow/blue colours, respectively. The initial cotton fabric (Co) was used to compare it with the treated fabrics. It can be seen that the values of a* and b* increase as an absolute value, with the values of a* being negative and b* being positive. This means that in the order H-NValC, Cl-NValC, and NO_2_-NValC, the colour becomes more saturated yellow-green, best expressed in the NO_2_-NValC fabric.

**Figure 2:**
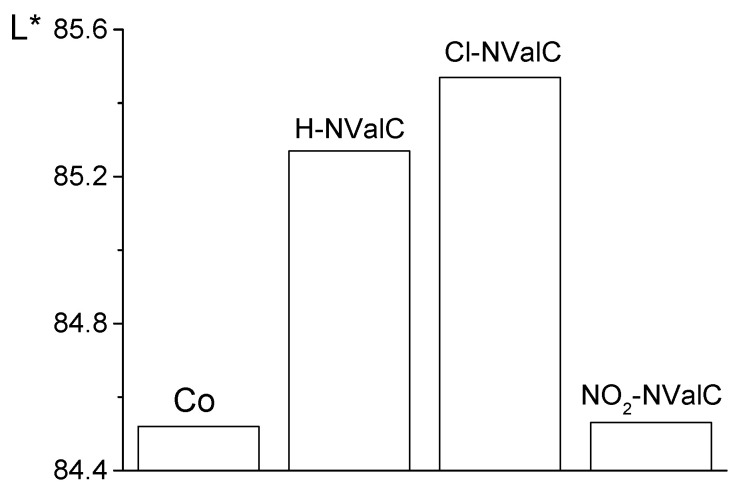
Lightness values L* of the pristine cotton fabric (Co) and of the fabrics modified with the respective peptides Cl-NValC, H-NValC, and NO_2_-NValC.

The lightness value, L*, is in the range of 1 ÷ 100 and defines white at 100 and black at 0, respectively. Figure 2 shows the lightness L* of fabrics compared to the cotton fabric (Co) used as standard. The Cl-NValC fabric is characterized by the greatest lightness, followed by the H-NValC fabric, while the L* values of the cotton fabric and the NO_2_-NValC fabric have approximately the same lower values.

**Figure 3:**
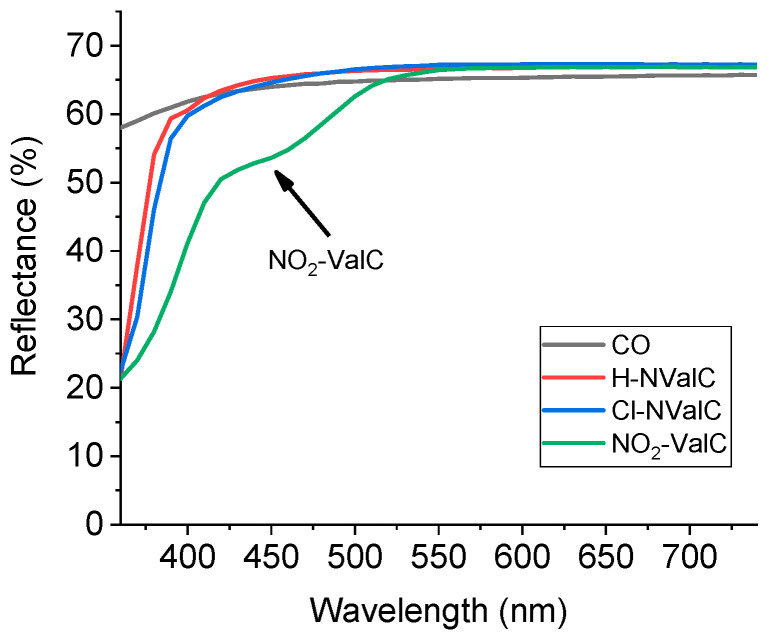
Reflectance spectra of the cotton fabric and of the fabrics modified with the respective peptides.

In Figure 3, the reflectance spectra of the three fabrics are compared with the spectrum of the original fabric, represented by a black line. It can be seen that the reflectance of the NO_2_-NValC fabric decreases from 350 nm to 500 nm, which corresponds to the yellow-green colour of the sample. For the other two fabrics, the reflectivity decreases outside the visible region of the spectrum, so they are white. There is a slight increase in their reflectance in the visible region of the spectrum, which is due to their fluorescence and the processing of the fabric during dyeing. There is a slight increase in their reflectance in the visible spectrum area after 400 nm due to their fluorescence and the fabric processing during dyeing.

### 2.5. Spectral Analysis of the H-NVal, Cl-NVal, and NO_2_-NVal Peptides in Ethanol Solution and After Their Deposition on Cotton Fabric

**Figure 4:**
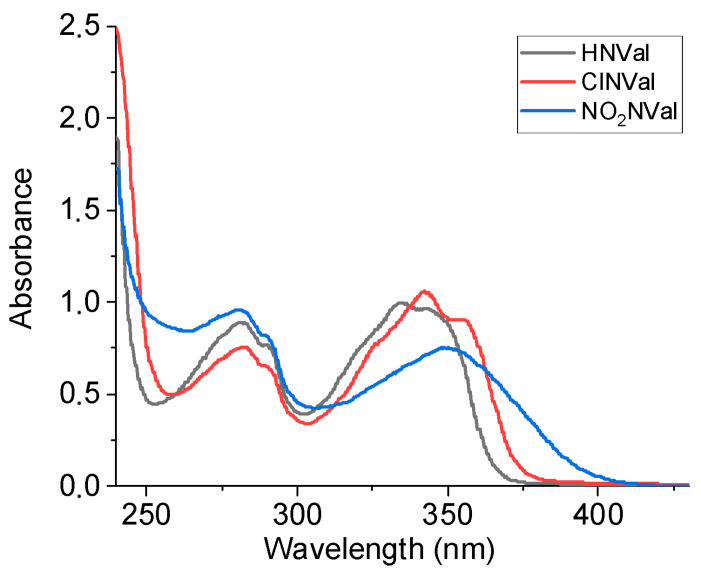
Absorption spectra of H-NVal, Cl-NVal, and NO_2_-NVal peptides in ethanol solution (c = 8.42 × 10^−5^ mol L^−1^).

The spectral properties of modified peptides with 1,8-naphthalimides, which have a hydrogen, chlorine atom, or nitro group as a substituent at the C-4 position, were investigated, and the data are summarized in Table 1. The measurements were carried out in an ethanol solution of the peptides, and a solid-state on the cotton fabrics was dyed with them. Due to the absence of a substituent with electron-donating properties, the polarization of the chromophore system in similar type 1,8-naphthalimides is weakly expressed. They absorb in the ultraviolet region at λ_A_ = 335 ÷ 350 nm (Figure 4), and the emitted fluorescence is blue colour with maxima at λ_F_ = 386 ÷ 396 nm and weak in intensity (Φ_F_ = 0.09–0.38 × 10^−2^) (Table 1). The calculated Stokes shift (ν_A_-ν_F_) is at 2930 ÷ 3944 cm^−1^ and depend on the polarization of 1,8-naphthalimide fluorophore.

**Figure 5:**
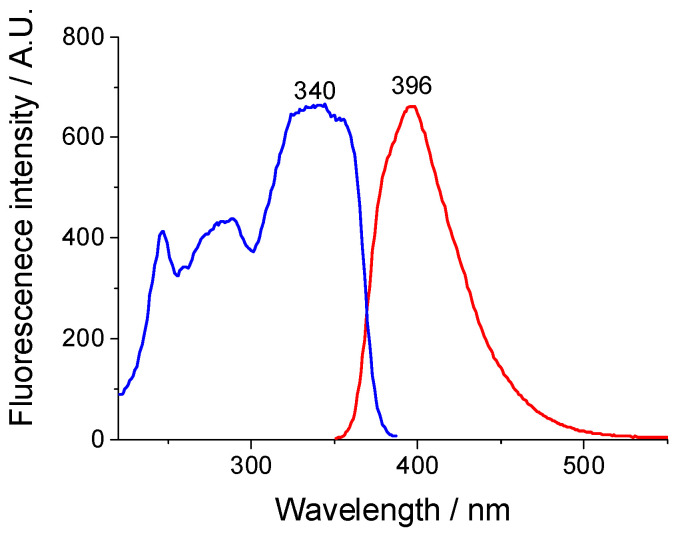
Excitation and fluorescence spectra of Cl-NVal in ethanol (c = 8.42 × 10^−5^ mol L^−1^).

Figure 5 shows the excitation (blue colour) and fluorescence (red colour) spectra of the Cl-NVal peptide in ethanol solution. The figure shows a well-defined fluorescence maximum at 396 nm, and the excitation spectrum has a maximum in the ultraviolet region at 340 nm. These values are typical for chloro-substituted 1,8-naphthalimides, indicating that incorporating 1,8-naphthalimides into the peptide molecule does not alter its fluorescence properties.

**Figure 6:**
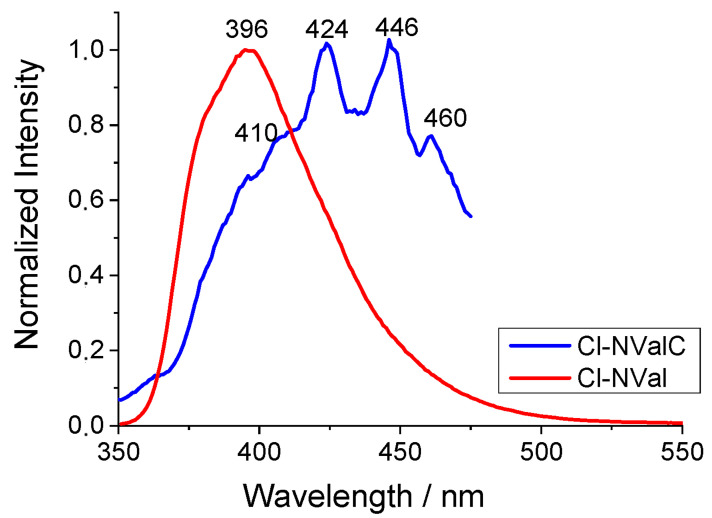
Fluorescence spectra of Cl-NVal in ethanol solution (c = 8.42 × 10^−5^ mol L^−1^) and Cl-NValC fabric.

In Figure 6, the fluorescence spectra of Cl-NVal in an ethanol solution (c = 8.42 × 10^−5^ mol L^−1^) and of cotton fabric dyed with it (Cl-NValC) are compared. The fluorescence spectrum of Cl-NValC exhibits a well-defined vibronic structure with maxima at 410 nm, 424 nm, 426 nm, and 460 nm, which are bathochromically shifted relative to those in solution. This shift is caused by the aggregation of the 1,8-naphthalimide fragments after deposition on the cotton surface induced by π-π stacking. On the other hand, the tight fixation of the chromophore system on the cotton surface limits the possibility of conformational changes.

**Figure 7:**
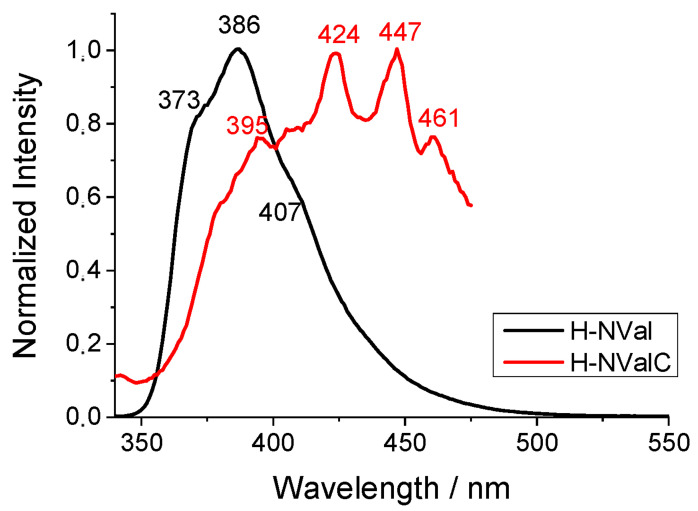
Fluorescence spectra of H-NVal in ethanol solution (c = 8.42 × 10^−5^ mol L^−1^) and H-NValC fabric.

Similar to Cl-NVal is the spectral behavior of H-NVal, where there is a hydrogen atom as a substituent in the C-4 position of the naphthalene nucleus (Figure 7). Since the peptide NO_2_-NVal contains an electron acceptor group, the observed fluorescence is insignificant.

### 2.6. Determination of Released Singlet Oxygen by Studying the Photooxidation of Potassium Iodide

The iodometric method, which requires a photosensitizer, light, and an oxygen medium, was used to determine the released singlet oxygen ^1^O_2_ [33,34]. We used this method to establish the released singlet oxygen during the irradiation of cotton fabrics treated with fluorescent peptides. In this case, the peptides modified with 1,8-naphthalimides are photosensitizers that, under the action of visible light, form singlet oxygen, which reacts with the colorless I^−^ to give the yellow compound I^3−^. Thus, the singlet oxygen-formation reaction can be followed by UV-visible spectrometry.

**Figure 8:**
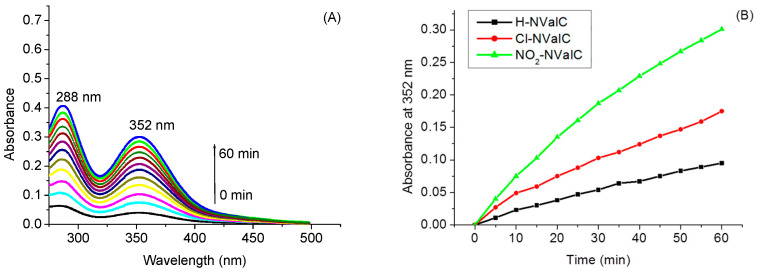
(**A**) Absorption spectra of photooxidation of KI to I^3−^ in the presence of NO_2_-NValC fabric as a function of visible light irradiation time; (**B**) Dependence of absorbance at 352 nm on time, upon irradiation of H-NValC, Cl-NValC, and NO_2_-NValC fabrics with visible light.

The absorption dependence as a function of the irradiation time of the solution containing the NVal fabric is visually represented in Figure 8A. The appearance of two peaks in the absorption spectrum at 288 nm and 352 nm, with increasing intensity over time, is a clear indicator of the rising concentration of I^3−^, which indicates the growing concentration of ^1^O_2_. A similar trend is observed for the other fabrics we investigated. The dependence of the absorbance at λ_A_ = 352 nm as a function of the irradiation time for the three investigated fabrics, H-NValC, Cl-NValC, and NO_2_-NValC, is presented in Figure 8B. It can be seen that the absorbance increases fastest for NO_2_-NValC fabric, followed by Cl-NValC fabric, and is slowest for H-NValC fabric. No absorbance was observed from the unirradiated KI solution, the peptides, and the cotton fabrics treated with them in the dark.

### 2.7. Antibacterial Assay of New Peptides in Solution and on the Cotton Fabric and Their Photoactivity

The research investigated the impact of light on the antimicrobial activity of the studied peptides in solution and on cotton fabric. The experiment, conducted in a liquid medium against the model bacteria *B. cereus* and *P. aeruginosa*, revealed distinct activity of the compounds based on the attached naphtalimide fluorophor and strain types. Negative controls (MPB and inoculum, without peptides) and positive controls (peptides and MPB, without inoculum, and DMSO as solvent) have been used to clarify the activity of peptides and treated cotton fabrics with them.

#### 2.7.1. Antibacterial Photoactivity of New Peptides in Solution

**Figure 9:**
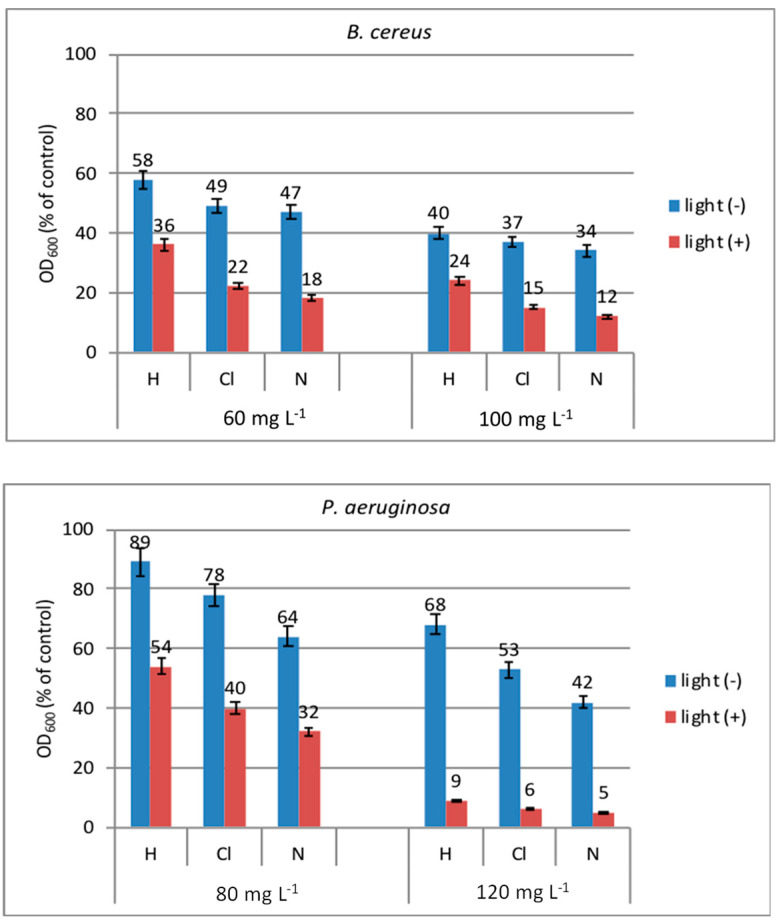
Effect of visible light on the antimicrobial activity of the new photoactive peptides entitles H-NVal as (H), Cl-NVal as (Cl), and NO_2_-NVal as (N) against the model strains *B. cereus* and *P. aeruginosa*.

The antibacterial activity of the dissolved peptide in the dark and under illumination with visible light is shown in Figure 9. H-compound (H-NVal) as (H) and showed lower activity against the tested strains than Cl- (Cl-NVal) and N- (NO_2_-NVal) compounds. Slightly higher growth inhibition was observed by the N-compound than by the Cl-compound. Gram-negative *P. aeruginosa* showed higher resistance to the compounds than Gram-positive *B. cereus*, which can be explained by the differences in the cell wall structures of Gram-positive and Gram-negative bacterial cells [35]. As shown in Figure 8, the antimicrobial effect was better expressed after light irradiation than in dark. In the dark, at a concentration of 120 µg mL^−1^, about 32%, 47%, and 58% inhibition of the growth of *P. aeruginosa* was determined in the presence of H-, Cl-, and N-peptides, respectively. In contrast, no visible growth was observed after light irradiation in the presence of all compounds. The results show that the peptides and cotton fabrics treated with them exhibit antimicrobial activity in the dark due to their chemical structure. However, their activity increases many times upon exposure to light. Thus, a synergism of antimicrobial and photodynamic activity is observed.

The increased antimicrobial activity of the studied photoactive peptides after light irradiation can be attributed to their ability to bind to the bacterial membrane and generate highly reactive singlet oxygen (^1^O_2_) upon photostimulation [36]. These reactive species attack the external layer of the bacterial membrane through multi-target action, causing oxidative stress and irreparable damage to the cellular bacterial components, ultimately leading to their inactivation [37].

#### 2.7.2. Antibacterial Photoactivity of New Peptides on Cotton Fabric

**Figure 10:**
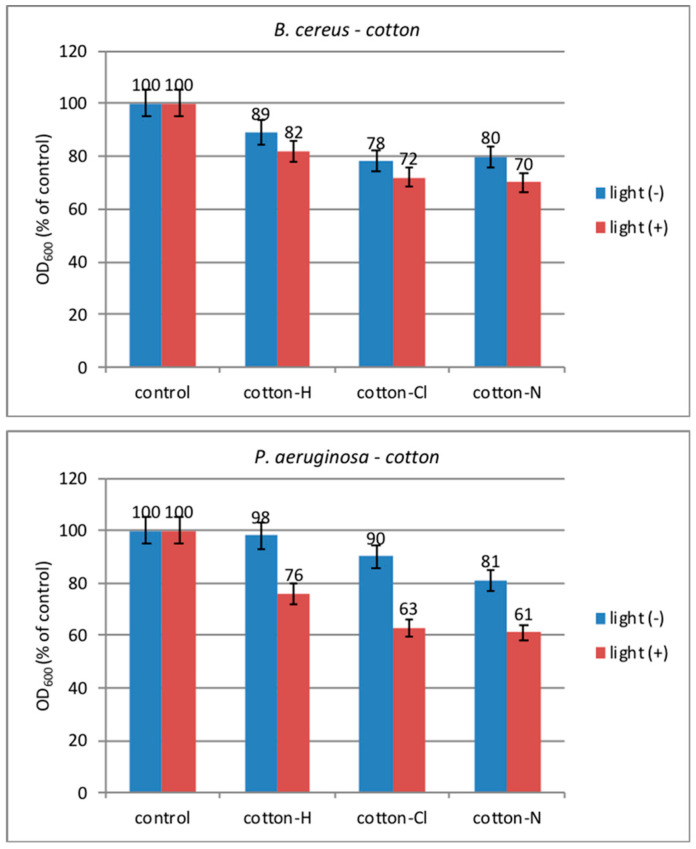
Effect of visible light on the antimicrobial activity of cotton fabric treated with H-, Cl-, and N-compounds against the model strains *B. cereus* and *P. aeruginosa*.

The antimicrobial modification of cotton surfaces is an alternative method for preventing the formation of highly resistant biofilms and can be achieved through various techniques [38]. As shown in Figure 10, cotton fabrics modified with the new H-, Cl-, and N-peptides inhibit the growth of the tested cultures compared to the native control sample. The activity slightly increased after light irradiation compared to that in the dark, and this effect was better expressed in the *P. aeruginosa* strain than in *B. cereus*. Gram-negative and Gram-positive bacteria display different sensitivities to pure singlet oxygen generated outside cells [39]. The outer membrane and lipopolysaccharide layer protect Gram-negative bacteria against environmental attacks, distinguishing them from Gram-positive bacteria. However, singlet oxygen can cause lipid peroxidation and subsequent radical-mediated chain reactions [40]. These free radicals are more aggressive to bacterium cells. When a photosensitizer is immobilized on a solid matrix like a textile, the main reason for bacteria inactivation is the generated singlet oxygen.

## 3. Experimental Part

### 3.1. Synthesis of the 1,8-Naphtalimide-β-alanine (***1***), 4-Chloro-1,8-naphtalimide-β-alanine (***2***), and 4-Nitro-1,8-naphtalimide-β-alanine (***3***)

3-(1,3-dioxo-1H-benzo[de]isoquinolin-2(3H)-yl)propanoic acid/1,8-naphtalimide-β-alanine (**1**), 3-(6-chloro-1,3-dioxo-1H-benzo[de]isoquinolin-2(3H)-yl)propanoic acid/(4-chloro-1,8-naphtalimide-β-alanine) (**2**), and 3-(6-nitro-1,3-dioxo-1H-benzo[de]isoquinolin-2(3H)-yl)propanoic acid/4-nitro-1,8-naphtalimide-β-alanine (**3**) have been synthesized by the described method [21], according to Scheme 1.

### 3.2. The Analytical Data for the Synthesized Peptides Prepared Were as Follows

#### 3.2.1. Compound H-NVal: (S)-5-Amino-4-((2S,3R)-2-((S)-2-((S)-1-((3-(1,3-dioxo-1H-benzo[de]isoquinolin-2(3H)-yl)propanoyl)-L-valyl-L-valyl-L-tyrosyl)pyrrolidine-2-carboxamido)-3-(1H-indol-3-yl)propanamido)-3-hydroxybutanamido)-5-oxopentanoic Acid (Figure 11)

White powder; >97% pure, m.p. 107–108 °C, t_R_ 32.12 min, HRMS (ESI) calculated for C_59_H_70_N_10_O_14_ [MH^+^]: 1142.5073; found: 1143.5119.

The optical rotation in methanol (c = 0.01) at 20 °C was −44°.

^1^H NMR (600 MHz, DMSO-*d*_6_), δ (ppm): 0.50–0.76 (m, 12H, 2-Val-CH_3_), 1.51 (d, *J* = 6.8 Hz, 1H, Thr-CH_3_), 1.64–1.69 (m, 2H, Pro-CH_2_), 2.02 (m, 2H, Glu-CH_2_), 2.04–2.28 (m, 4H, Pro-CH_2_; Glu-CH_2_), 2.27 (t, *J* = 7.1 Hz, 2H, Glu-CH_2_), 2.34 (t, *J* = 7.1 Hz, 2H, β-Ala-CH_2_), 2.66 (m, 2H, 2-Val-CH), 3.49 (d, *J* = 7.0 Hz, 2H, Trp-CH_2_), 3.78 (m, 4H, Pro-CH_2_; Tyr-CH_2_), 3.90 (t, *J* = 7.0 Hz, 1H, β-Ala-CH_2_), 3.92–3.97 (m, 5H, 2-Val-CH; Pro-CH; Glu-CH; Thr-CH), 3.98 (m, 1H, Thr-CH-OH), 4.02 (tt, *J* = 7.6, 5.1 Hz, 2H, Trp-CH; Tyr-CH), 6.38 (s, 1H, OH), 6.39–6.73 (m, 4H, Ar-Tyr), 6.74 (2H, 2-Val-NH), 6.78 (1H, Thr-NH), 6.80–6.97 (m, 4H, Ar-Trp), 6.82 (1H, Tyr-NH), 7.03–8.25 (m, 6H, Ar-naphtalimide), 7.11 (bs, 1H, β-Ala-NH), 7.25 (1H, Tyr-NH), 7.46 (1H, Tyr-NH), 10.46 (s, 1H, OH), 10.57 (s, 1H, OH).

^13^C NMR (151 MHz, DMSO-*d*_6_) δ 174.48, 173.63, 172.23, 172.07, 171.26, 171.02, 170.42, 170.36, 170.11, 163.75, 162.79, 156.25, 136.48, 134.75, 131.75, 131.13, 130.48, 128.14, 127.89, 127.66, 124.13, 122.59, 121.29, 118.84, 118.68, 115.38, 111.68, 110.24, 66.88, 59.89, 58.60, 58.30, 57.86, 54.24, 52.32, 47.20, 36.97, 36.26, 31.23, 31.21, 30.63, 27.55, 24.74, 19.83, 19.63, 18.31.

#### 3.2.2. Compound Cl-NVal: (S)-5-Amino-4-((2S,3R)-2-((S)-2-((S)-1-((3-(6-chloro-1,3-dioxo-1H-benzo[de]isoquinolin-2(3H)-yl)propanoyl)-L-valyl-L-valyl-L-tyrosyl)pyrrolidine-2-carboxamido)-3-(1H-indol-3-yl)propanamido)-3-hydroxybutanamido)-5-oxopentanoic Acid (Figure 12)

White powder; >97% pure, m.p. 112–113 °C, tR 36.40 min, HRMS (ESI) calculated for C_59_H_69_ClN_10_O_14_ [MH^+^]: 1176.4683; found: 1177.4728.

The optical rotation in methanol (c = 0.01) at 20 °C was −44°.

^1^H NMR (600 MHz, DMSO-*d*_6_), δ (ppm): 0.73–0.98 (m, 12H, 2-Val-CH_3_), 1.74 (d, *J* = 6.8 Hz, 1H, Thr-CH_3_), 1.92–2.03 (m, 2H, Pro-CH_2_), 2.24 (m, 2H, Glu-CH_2_), 2.51–2.56 (m, 4H, Pro-CH_2_; Glu-CH_2_), 2.57 (t, *J* = 7.1 Hz, 2H, Glu-CH_2_), 2.74 (t, *J* = 7.1 Hz, 2H, β-Ala-CH_2_), 2.89 (m, 2H, 2-Val-CH), 4.14 (d, *J* = 7.0 Hz, 2H, Trp-CH_2_), 4.16 (m, 4H, Pro-CH_2_; Tyr-CH_2_), 4.18 (t, *J* = 7.0 Hz, 1H, β-Ala-CH_2_), 4.20–4.23 (m, 5H, 2-Val-CH; Pro-CH; Glu-CH; Thr-CH), 4.25 (m, 1H, Thr-CH-OH), 4.52 (tt, *J* = 7.6, 5.1 Hz, 2H, Trp-CH; Tyr-CH), 6.60 (s, 1H, OH), 6.61–6.63 (m, 4H, Ar-Tyr), 6.96 (2H, 2-Val-NH), 7.02 (1H, Thr-NH), 7.03–7.05 (m, 4H, Ar-Trp), 7.17 (1H, Tyr-NH), 7.19 (bs, 1H, β-Ala-NH), 7.25 (1H, Tyr-NH), 7.45 (1H, Tyr-NH), 7.67–8.58 (m, 5H, Ar-naphtalimide), 10.49 (s, 1H, OH), 10.58 (s, 1H, OH).

^13^C NMR (151 MHz, DMSO-*d*_6_) δ 174.47, 173.64, 172.20, 172.07, 171.26, 170.45, 170.32, 170.12, 163.32, 162.80, 156.27, 137.89, 136.51, 131.99, 131.28, 130.48, 129.06, 128.85, 128.14, 127.90, 124.13, 123.29, 118.83, 118.68, 115.29, 110.26, 66.87, 59.90, 58.61, 58.32, 52.34, 37.09, 33.63, 31.23, 30.64, 30.48, 27.55, 19.82, 19.62, 18.75, 18.50, 18.30.

#### 3.2.3. Compound NO_2_-NVal: (S)-5-Amino-4-((2S,3R)-2-((S)-2-((S)-1-((3-(6-nitro-1,3-dioxo-1H-benzo[de]isoquinolin-2(3H)-yl)propanoyl)-L-valyl-L-valyl-Ltyrosyl) pyrrolidine-2-carboxamido)-3-(1H-indol-3-yl)propanamido)-3-hydroxybutanamido)-5-oxopentanoic Acid (Figure 13)

White powder; >97% pure, m.p. 116–117 °C, tR 33.95 min, HRMS (ESI) calculated for C_59_H_69_N_11_O_16_ [MH^+^]: 1187.4924; found: 1188.4968.

The optical rotation in methanol (c = 0.01) at 20 °C was −40°.

^1^H NMR (600 MHz, DMSO-*d*_6_), δ (ppm): 0.73–1.04 (m, 12H, 2-Val-CH_3_), 1.74 (d, *J* = 6.8 Hz, 1H, Thr-CH_3_), 1.88–1.92 (m, 2H, Pro-CH_2_), 2.26 (m, 2H, Glu-CH_2_), 2.55–2.58 (m, 4H, Pro-CH_2_; Glu-CH_2_), 2.59 (t, *J* = 7.1 Hz, 2H, Glu-CH_2_), 2.74 (t, *J* = 7.1 Hz, 2H, β-Ala-CH_2_), 2.89 (m, 2H, 2-Val-CH), 4.13 (d, *J* = 7.0 Hz, 2H, Trp-CH_2_), 4.16 (m, 4H, Pro-CH_2_; Tyr-CH_2_), 4.18 (t, *J* = 7.0 Hz, 1H, β-Ala-CH_2_), 4.20–4.22 (m, 5H, 2-Val-CH; Pro-CH; Glu-CH; Thr-CH), 4.25 (m, 1H, Thr-CH-OH), 4.27 (tt, *J* = 7.6, 5.1 Hz, 2H, Trp-CH; Tyr-CH), 6.60 (s, 1H, OH), 6.61–6.62 (m, 4H, Ar-Tyr), 6.95 (2H, 2-Val-NH), 7.02 (1H, Thr-NH), 7.03–7.05 (m, 4H, Ar-Trp), 7.17 (1H, Tyr-NH), 7.19 (bs, 1H, β-Ala-NH), 7.29 (1H, Tyr-NH), 7.54 (1H, Tyr-NH), 7.67–8.71 (m, 5H, Ar-naphtalimide), 10.64 (s, 1H, OH), 10.77 (s, 1H, OH).

^13^C NMR (151 MHz, DMSO-*d*_6_) δ 174.47, 173.63, 172.07, 171.01, 170.45, 170.28, 170.12, 163.28, 162.80, 162.49, 156.26, 149.58, 136.50, 132.12, 130.56, 130.48, 130.03, 128.79, 127.90, 127.16, 124.71, 123.29, 121.21, 118.68, 115.40, 110.26, 66.88, 59.91, 58.33, 57.89, 52.56, 52.35, 36.25, 33.55, 31.24, 30.64, 30.47, 27.56, 24.74, 19.81, 19.62, 18.76, 18.58.

### 3.3. Cotton Fabric Modification with New Peptides H-NVal, Cl-NVal, and NO_2_-NVal

Cotton fabric samples were soaked with the peptides’ ethanol solution at a concentration of 0.5% by weight of the fabric and a liquor-to-fabric ratio of 3:1. The fabrics were allowed to air dry at room temperature and then rinsed with water and a detergent solution (Lavoral S 313; Bozzeto group) with concentration 2 g L^−1^.

### 3.4. Color Measurements of the Fabrics Modified with Peptides

The fabrics’ color characteristics were determined using a Konica Minolta optoelectronic system with a CM-36dG spectrophotometer, a D_65_ light source, and D/10° illumination-observation geometry. The reflectance spectra in the visible region (R%) and the colour coordinates L*a*b* were used for characterization.

### 3.5. Fluorescent Properties of Peptides and Cotton Fabrics

Excitation and fluorescence spectra were taken with a Cary Eclipse (Agilent, New York, NY, USA) spectrophotometer in the range 350 ÷ 550 nm, with a resolution of 0.5 nm and double-grating monochromators for excitation and emission.

The new peptides H-NVal, Cl-NVal, and NO_2_-NVal fluorescent spectra were measured in ethanol (C = 8.42 × 10^−5^ mol L^−1^). The fabric modified with peptides was measured in a dry state. A sample of each fabric (10 mm × 30 mm) was placed diagonally in a quartz cuvette. Quinine bisulfate 1N H_2_SO_4_ (Φ_F_ = 0.546) has been used as a standard for the calculation of the fluorescence quantum yield of peptides.

### 3.6. Determination of the Released Singlet Oxygen from the Fabrics

A sample of the investigated fabrics (10 mm × 10 mm) was immersed in 5 mL of KI solution with a concentration of 0.5 M and irradiated with a lamp emitting visible light. The distance between the lamp and the sample was 10 cm. Every 5 min, a spectrum of the solution was taken in the range 270 ÷ 500 nm. An INESA, LED, 1521 LUMEN, 14 W, daylight/6500 K lamp was used as a visible light source. An ONDA Spectrophotometer UV-31 SCAN, 190 ÷ 1100 nm, UV-Vis spectrophotometer was used to record the absorption spectra.

## 4. Conclusions

Three new peptides, derivatives of the natural Valorphin, were synthesized and modified with 1,8-naphthalimide fluorophores. The peptides were deposited on cotton fabric by their ethanol solutions. The different substituents in fluorophore structure influence the colour characteristics of the textile. The colour coordinates of the NO_2_-NValC fabric are in the yellow-green region, distinguishing it from the other fabrics (H-NValC and Cl-NValC), which act as optical brighteners for the pristine fabric. Fluorescence analysis of the peptides in solution showed that they have very well-defined maxima in the excitation and fluorescence spectra’s ultraviolet region, characteristic of the 1,8-naphthalimide fluorophore. Upon deposition on cotton fabric, a well-defined vibronic structure is observed in the fluorescence spectrum with several maxima that are bathochromically shifted relative to those in solution. It is likely due to the arrangement and aggregation of the 1,8-naphthalimide fragments after their deposition on the cotton surface, as well as the firm fixation and limitation of the possibility of conformational changes of the peptides.

The antibacterial assay of new peptides in the solution shows promising activity against model Gram-positive and Gram-negative bacteria. The inclusion of a luminophore in the peptide structure allows the use of light as a factor to enhance their antimicrobial efficacy.

This trend was also observed for the antibacterial activity of the peptides-modified fabrics. A nitro substituent in the fluorophore structure enhanced the peptide and corresponding fabric (NO_2_-NValC) activities. With their antibacterial potential, the peptides are more effective in solution than on the fibre surface, as their mobility is essential for good contact with bacteria.

## Figures and Tables

**Figure 11:**
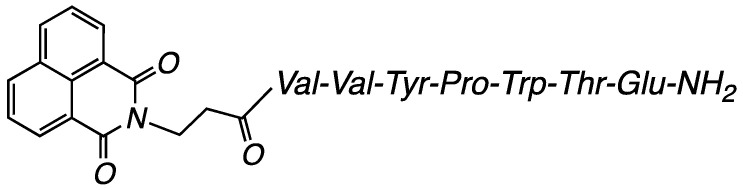
Peptide H-NVal.

**Figure 12:**
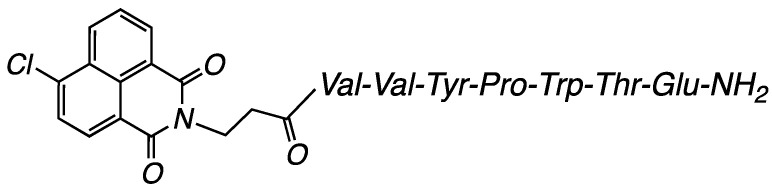
Peptide Cl-NVal.

**Figure 13:**
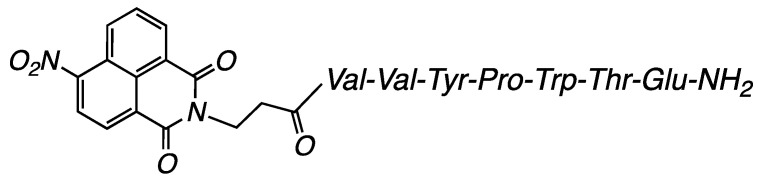
Peptide NO_2_-NVal.

**Table 1:** Photophysical characteristics of peptides in ethanol solution solvents.

Peptides	λ_A_ (nm)	ε (mol L^−1^ cm^−1^)	λ_F_ (nm)	ν_A_-ν_F_ (cm^−1^)	Φ_F_
H-NVal	335,343	11,850	386	3944	0.0038
Cl-NVal	343,355	12,510	396	3902	0.0021
NO_2_-NVal	350	8810	390	2930	0.0009

## Data Availability

Data will be made available on request.

## Supplementary Materials

The following supporting information can be downloaded at: https://www.mdpi.com/article/10.3390/molecules29225421/s1, Materials and apparatus, General procedure for the peptide synthesis of compounds (H-NVal, Cl-NVal, NO_2_-NVal) and In vitro antimicrobial assay Figure S1: Synthetic pathway of the new 1,8-naphthalimide-conjugated hemorphin derivatives (H-NVal, Cl-NVal, NO_2_-NVal); Figure S2: ^1^H and ^13^C- NMR spectra of H-NValC, Cl-NValC, and NO_2_-NValC peptides, Figure S3: NMR spectra of compound H-NVal: COSY (left) and NOESY (right).
