# Supplementary material for: Novel Peptide Analogues of Valorphin-Conjugated 1,8-Naphthalimide as Photodynamic Antimicrobial Agent in Solution and on Cotton Fabric
Source: Molecules. 2024 Nov 17;29(22):5421. doi: 10.3390/molecules29225421 (PMC11597154; doi:10.3390/molecules29225421)
Supplement: Supplementary file 1 [file molecules-29-05421-s001.zip › molecules-3295959-supplementary.pdf]

molecules

MDPI

Article

Novel peptide analogues of valorphin conjugated 1,8-naphthalimide as photodynamic antimicrobial agent in solution and on cotton fabric

Desislava Staneva [1*], Petar Todorov [2], Stela Georgieva [3], Petia Peneva [2], and Ivo Grabchev [4,*]

[1] University of Chemical Technology and Metallurgy, Department of Textile, Leathers and Fuels, Sofia, Bulgaria; e-mail: grabcheva@mail.bg
[2] University of Chemical Technology and Metallurgy, Department of Organic Chemistry, Sofia, Bulgaria;
[3] University of Chemical Technology and Metallurgy, Department of Analytical Chemistry, Sofia, Bulgaria;
[4] Sofia University "St. Kliment Ohridski", Faculty of Medicine, Sofia, Bulgaria;

* Correspondence: grabcheva@mail.bg; D.S. and i.grabchev@chem.uni-sofia.bg I.G

Materials and apparatus

All reagents and solvents were analytical or HPLC grade and were bought from Fluka or Merck, and used without further purification. The protected amino acids and Fmoc (9-fluorenylmethoxycarbonyl)-Rink Amide MBHA (4-methylbenzhydrylamine) Resin were purchased from Iris Biotech (Germany). The 3-functional amino acids were embedded as follows: Glu – as Fmoc-Glu(tBu)-OH, Tyr – as Nα-Fmoc-Tyr(tBu)-OH, Thr - as Nα-Fmoc-Thr(t-Bu)-OH, and Trp – as Nα-Fmoc-Trp(Boc)-OH. The molecular mass and purity of the compound were confirmed by high-resolution electrospray mass spectrometry on a Q Exactive high-resolution mass spectrometer (Thermo Fisher Scientific Inc., USA) equipped with TurboFlow TM Transcend chromatography system (Thermo Fisher Scientific Inc., USA) and heated electrospray ionization (HESI II) source. Data acquisition and processing were done by XCalibur® 2.4 software (Thermo Fisher Scientific Inc., USA). The instrumental parameters were as follows: Spray Voltage – 4.0 KV, Sheath Gas – 30 AU, Auxiliary Gas – 12 AU, Capillary Temperature – 300°C, Spare Gas – 3 AU, Heater Temperature – 300°C. Full scan experiments were carried out in a range of 120 – 2 000 m/z at 140 000 resolution. Optical rotations were recorded on a MCP200 modular circular polarimeter (Anton Paar Opto Tec GmbH, Seelze, Germany).

The NMR spectra were recorded on a Bruker Avance II+ spectrometer operating with frequency 600 MHz for ^1H and 125 MHz for ^{13}C in DMSO-d6. Chemical shifts δ are reported in ppm, and coupling constants J are reported in Hz. The precise assignment of the ^1H and ^{13}C NMR spectra was accomplished by the measurement of 2D homonuclear correlation (COSY, NOESY).

General procedure for the peptide synthesis of compounds (H-NVal, Cl-NVal, NO_2-NVal)

All N-modified peptide analogs of valorphin were synthesized manually by the solid-phase method using Fmoc-strategy. Peptides are synthesized on Fmoc-Rink-Amide MBHA resin (loading 0.71 mmol/g resin; cross-linking 1% DVB; 100-200 mesh). TBTU (2-(1H-benzotriazole-1-yl)-1,1,3,3-tetramethylaminiumtetrafluoro- borate) was used as an efficient peptide coupling reagent. HOBt (1-hydroxybenzotriazole hydrate) and DIEA (N,N-diisopropylethylamine) were used for amino acid activation. Peptide chains were elongated in consecutive cycles of deprotection and coupling. The coupling reactions were performed using amino acid/TBTU/HOBt/DIEA/resin with a molar ratio of 3/2.9/3/6/1, in a 1:1 mixture of DMF/DCM. The Fmoc group at every step was deprotected by treatment with 20% piperidine solution in N,N-dimethylformamide (DMF). After each reaction step, the resin was washed with DMF (3 x 1 min), isopropyl alcohol (3 x 1 min), and CH2Cl2 (3 x 1 min). The coupling and deprotection reactions were monitored by the standard Kaiser

test. The cleavage of the final synthesized peptides from the resin was performed using a mixture of 95% trifluoroacetic acid (TFA), 2.5% triisopropylsilane (TIS), and 2.5% distilled water. The peptides were obtained as a filtrate in TFA and precipitated with cold dry diethyl ether. The precipitate was filtered, dissolved in water, and lyophilized to yield the compounds as a powder. The crude peptides were dissolved in H2O and acetonitrile was added until complete dissolving was observed. The peptides were obtained as white powders with a purity of >97% as determined by analytical HPLC. The structures were confirmed by high-resolution electrospray mass spectrometry and NMR spectroscopy. Peptide purity was monitored on a reversed-phase high-performance liquid chromatography (RP-HPLC), column: SymmetryShield™ RP-18, 3.5µ, (50 x 4.6 mm), flow: 1 mL min^{-1}, H_2O (0.1% TFA)/CH_3CN (0.1% TFA), gradient 0→100 % (45 min) and 100% (5 min). The crude peptides were purified by semi-preparative HPLC on column XBridge™ Prep C18 10µm (10 x 250 mm), flow: 5 mL min^{-1} H_2O (0.1% TFA)/CH_3CN (0.1% TFA), gradient 20→100% (50 min). Melting points were monitored on a standard Kofler hot-stage microscope.

In vitro antimicrobial assay

The antimicrobial activity of the investigated compounds and textile materials was tested against Gram-positive Bacillus cereus and Gram-negative Pseudomonas aeruginosa, which were used as model bacterial strains (Collection of the Institute of Microbiology, Bulgarian Academy of Sciences). Microbial cultures were maintained at 4 °C on meat-peptone agar (MPA) slants and transferred monthly.

A broth dilution assay was performed to evaluate quantitatively the antimicrobial activity of the peptides against the model strains. The compounds were dissolved in DMSO at a started concentration of 2.0 mg mL^{-1} and further diluted in test tubes with meat-peptone broth (MPB, pH=7.0) to final concentrations in the range of 10÷150 µg mL^{-1}. Inocula were prepared by diluting the overnight cultures with 0.9% NaCl to a 0.5 McFarland standard. After inoculation with each standardized microbial suspension, the tubes were incubated at the appropriate temperature for 18 h in light and dark. In parallel, positive controls (compound and MPB, without inoculum) and negative controls (MPB and inoculum, without compounds) were prepared. The optical density of each tube was read at 600 nm (OD600) as a measure of microbial growth. The % microbial growth was determined based on the positive control, which was considered 100%. All assays were performed in triplicate, and the averages were taken (standard deviations are less than 5%).

The antimicrobial activity of cotton fabrics treated with the peptides was tested in MPB against the model strains. Tubes containing MPB and square cotton specimens (10 mm x 10 mm) were inoculated with each standardized microbial suspension. Tubes with native cotton fabric and without specimens were also prepared as controls. Two sets of tubes were ready for the tests in the presence and the absence of light. After 18 h incubation at the 37^0C, the specimens were removed, and OD600 was determined. The antimicrobial activity of the samples was evaluated by the reduction of bacterial growth in the presence of the treated specimens compared to the native. The tests were done in triplicate, and mean values were given (standard deviations were less than 5%).

S1. Synthetic pathway

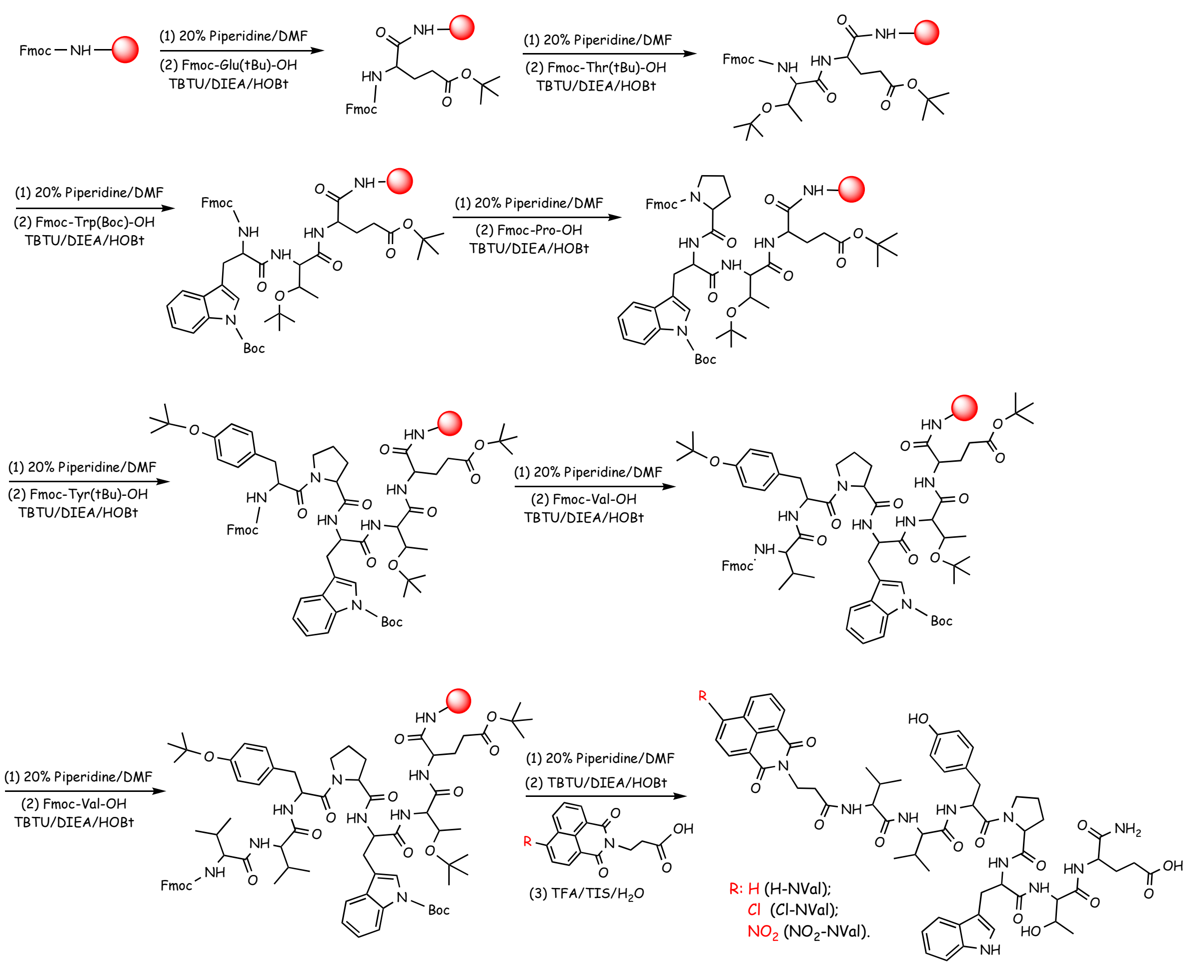

Figure S1. Synthetic pathway of the new 1,8-naphthalimide-conjugated hemorphin derivatives (**H-NVal, Cl-NVal, NO_2-NVal**)

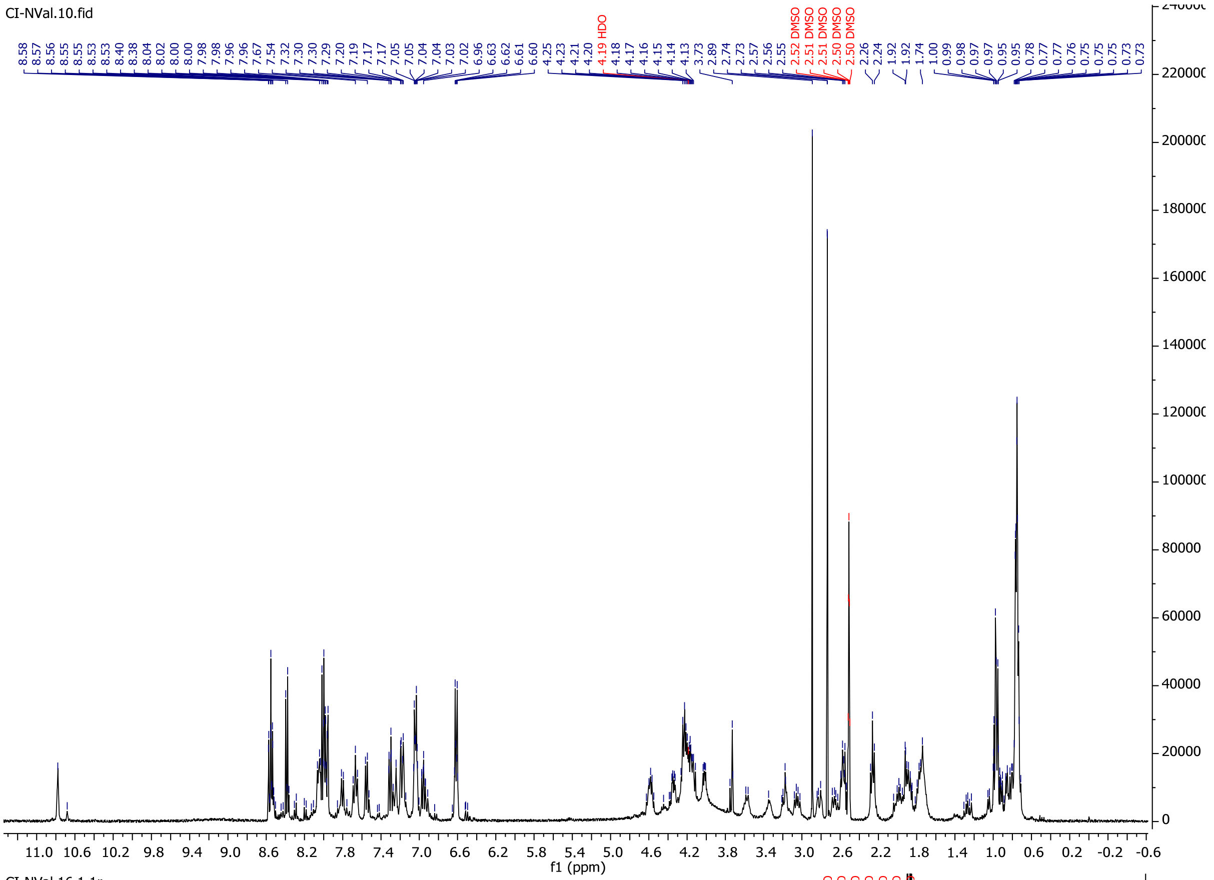
CI-NVal.10.fid
8.58 8.57 8.56 8.55 8.55 8.53 8.53 8.40 8.38 8.04 8.02 8.00 8.00 7.98 7.98 7.96 7.96 7.67 7.54 7.32 7.30 7.30 7.29 7.20 7.19 7.17 7.05 7.04 7.04 7.03 7.02 6.96 6.63 6.62 6.61 6.60 4.25 4.23 4.21 4.20
4.19 HDO
4.18 4.17 4.16 4.15 4.14 4.13 3.73 2.89 2.74 2.73 2.57 2.56 2.55
2.52 DMSO
2.51 DMSO
2.51 DMSO
2.50 DMSO
2.50 DMSO
2.26 2.24 1.92 1.92 1.74 1.00 0.99 0.98 0.97 0.97 0.95 0.95 0.78 0.77 0.77 0.76 0.75 0.75 0.75 0.73 0.73
f1 (ppm)

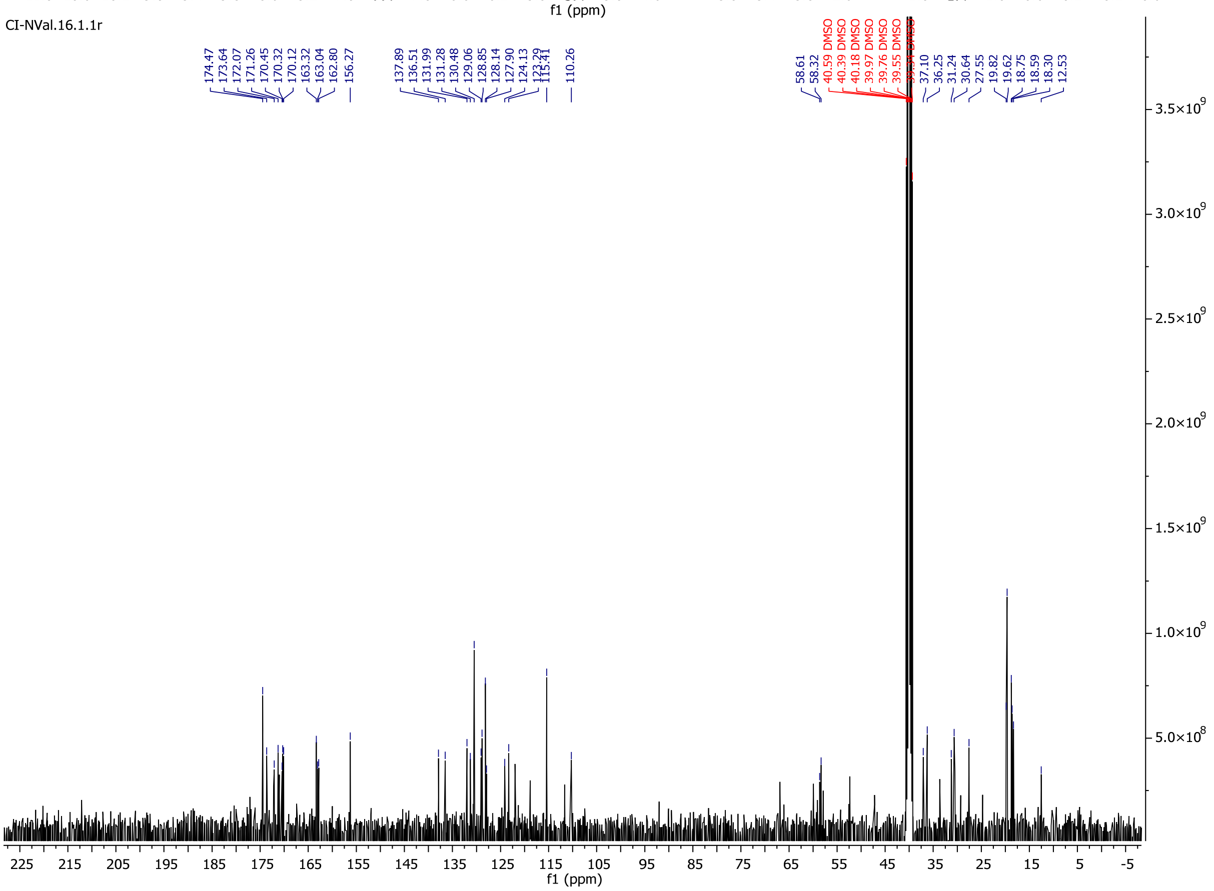
CI-NVal.16.1.1r
174.47 173.64 172.07 171.26 170.45 170.32 170.12 163.32 163.04 162.80 156.27
137.89 136.51 131.99 131.28 130.48 129.06 128.85 128.14 127.90 124.13 123.29 115.41 110.26
58.61 58.32
40.59 DMSO
40.39 DMSO
40.18 DMSO
39.97 DMSO
39.76 DMSO
39.55 DMSO
39.34 DMSO
37.10 36.25 31.24 30.64 27.55 19.82 19.62 18.75 18.59 18.30 12.53
f1 (ppm)

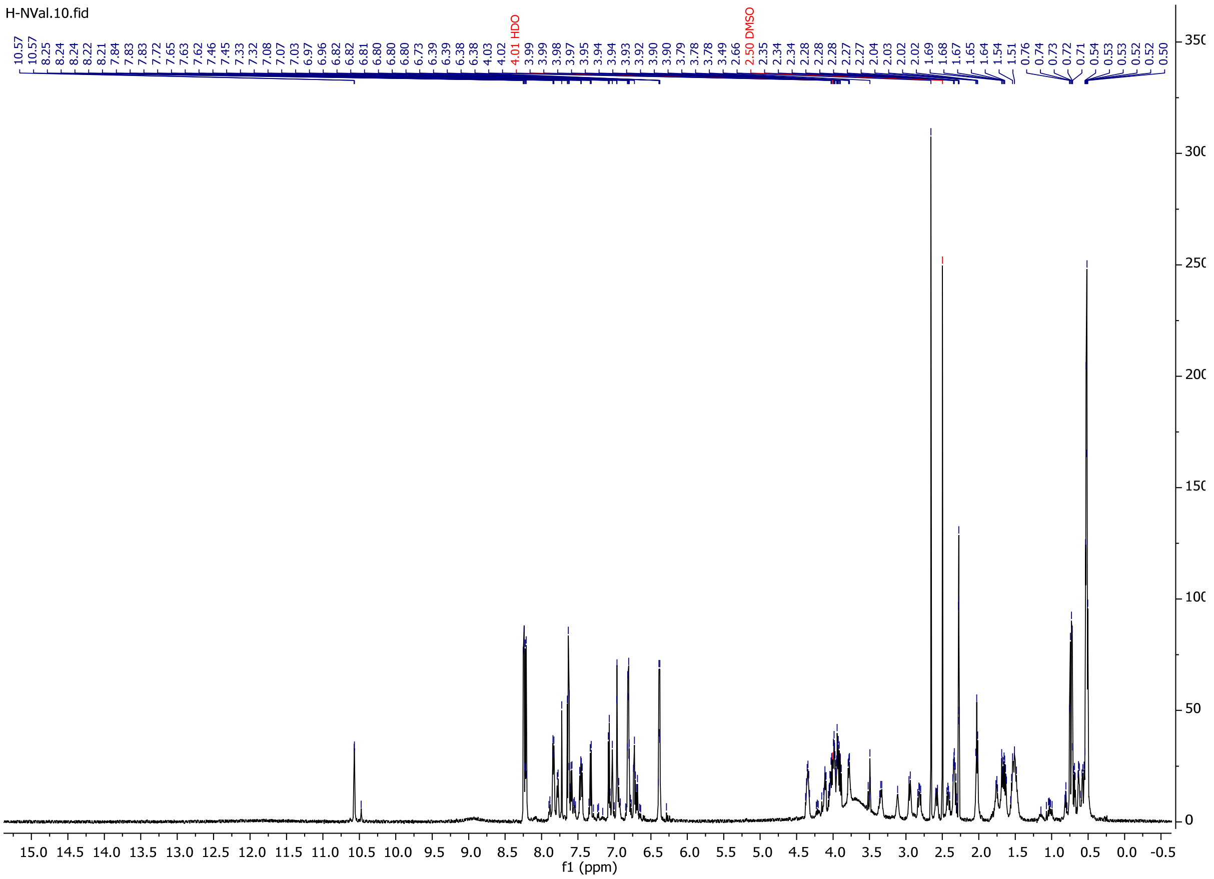
H-NVal.10.fid
10.57 10.57 8.25 8.24 8.24 8.22 8.21 7.84 7.83 7.83 7.72 7.65 7.63 7.62 7.46 7.45 7.33 7.32 7.08 7.07 7.03 6.97 6.96 6.82 6.82 6.81 6.80 6.80 6.80 6.73 6.39 6.39 6.38 6.38 4.03 4.02
4.01 HDO
3.99 3.99 3.98 3.97 3.95 3.94 3.94 3.93 3.92 3.90 3.90 3.79 3.78 3.78 3.49 2.66
2.50 DMSO
2.35 2.34 2.34 2.28 2.28 2.28 2.27 2.27 2.04 2.03 2.02 2.02 1.69 1.68 1.67 1.65 1.64 1.54 1.51 0.76 0.74 0.73 0.72 0.71 0.54 0.53 0.53 0.52 0.52 0.50
f1 (ppm)

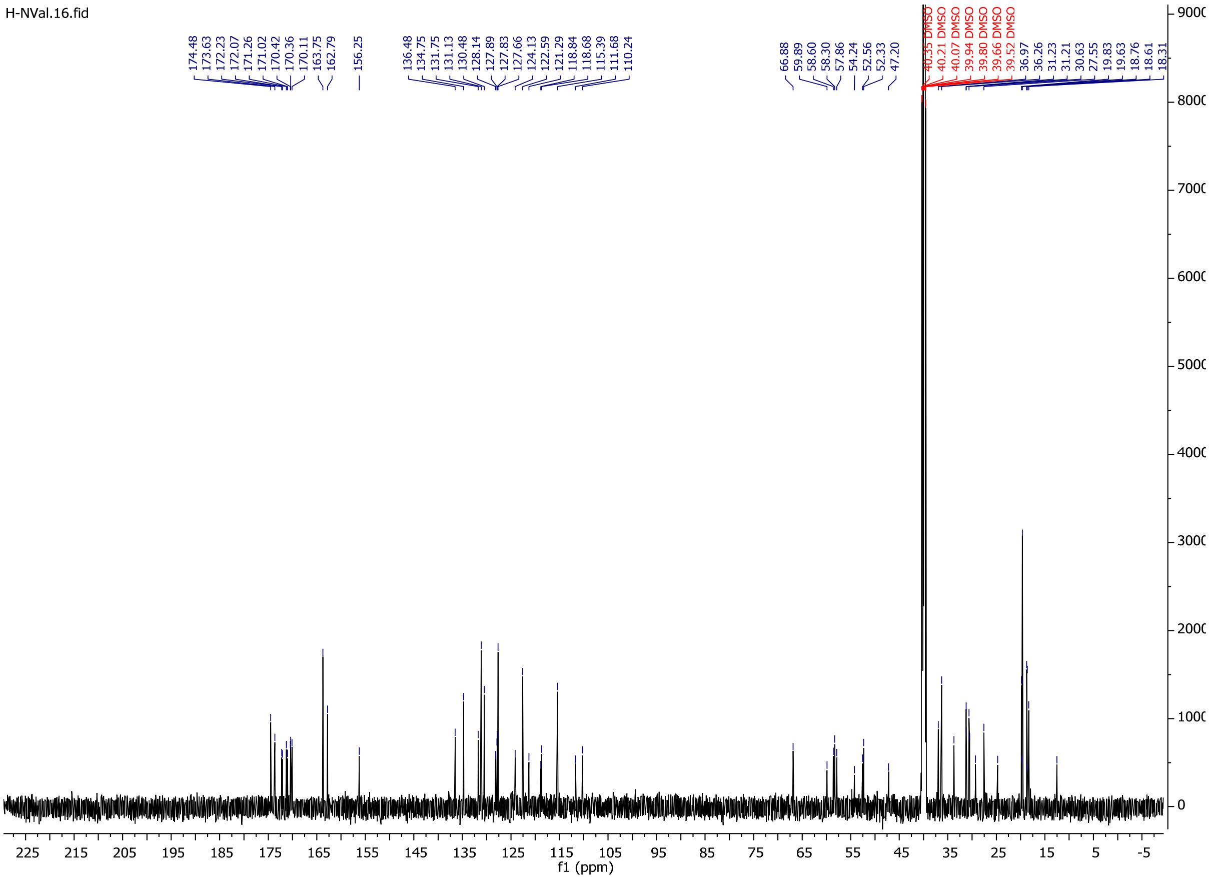
H-NVal.16.fid
174.48 173.63 172.23 172.07 171.26 171.02 170.42 170.36 170.11 163.75 162.79 156.25 136.48 134.75 131.75 131.13 130.48 128.14 127.89 127.83 127.66 124.13 122.59 121.29 118.84 118.68 115.39 111.68 110.24 66.88 59.89 58.60 58.30 57.86 54.24 52.56 52.33 47.20
40.35 DMSO
40.21 DMSO
40.07 DMSO
39.94 DMSO
39.80 DMSO
39.66 DMSO
39.52 DMSO
36.97 36.26 31.23 31.21 30.63 27.55 19.83 19.63 18.76 18.61 18.31
f1 (ppm)

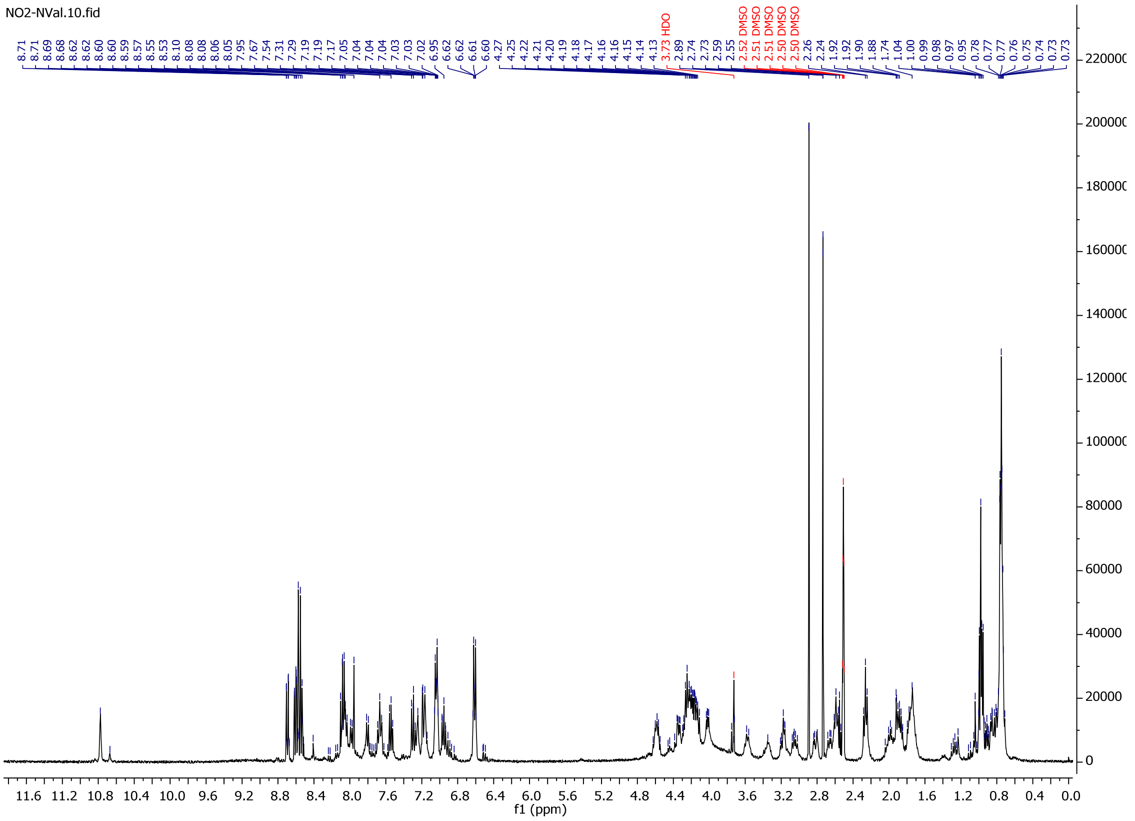

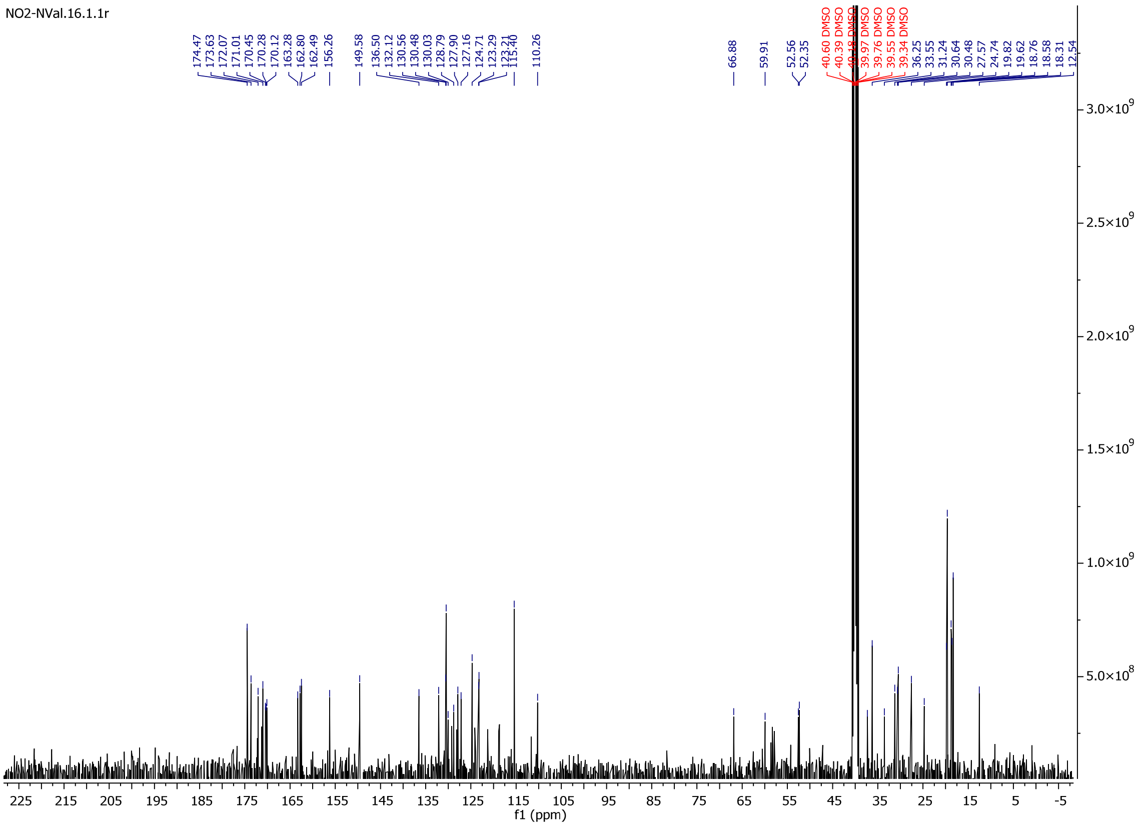

Figure S2. ^{1}H and ^{13}C- NMR spectra of H-NValC, Cl-NValC, and NO_2-NValC peptides

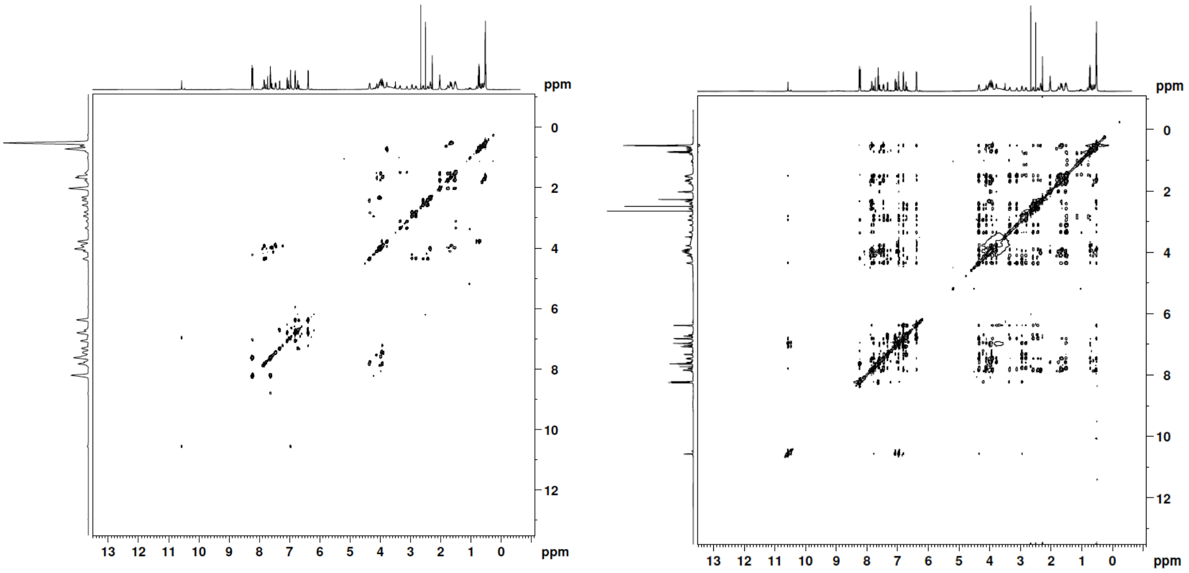

Figure S3. NMR spectra of compound **H-NVal**: COSY (left) and NOESY (right).
